# Reliability of continuous vital sign monitoring in post-operative patients employing consumer-grade fitness trackers: A randomised pilot trial

**DOI:** 10.1177/20552076241254026

**Published:** 2024-05-13

**Authors:** Philipp Helmer, Sebastian Hottenrott, Kathrin Wienböker, Rüdiger Pryss, Vasileios Drosos, Anna Katharina Seitz, Daniel Röder, Aleksandar Jovanovic, Jürgen Brugger, Peter Kranke, Patrick Meybohm, Bernd E Winkler, Michael Sammeth

**Affiliations:** 1 Department of Anaesthesiology, Intensive Care, Emergency and Pain Medicine, University Hospital Würzburg, Würzburg, Germany; 2 Institute for Clinical Epidemiology and Biometry, University Hospital Würzburg, Würzburg, Germany; 3 Department of Thoracic and Cardiovascular Surgery, University Hospital Würzburg, Würzburg, Germany; 4 Department of Urology and Pediatric Urology, University Hospital Würzburg, Würzburg, Germany; 5 Department of Applied Sciences and Health, Coburg University of Applied Sciences and Art, Coburg, Germany

**Keywords:** Health tracker, smartwatch, wearables, Garmin Fenix 6 pro, Apple Watch 7, Withings ScanWatch

## Abstract

**Introduction:**

Fitness trackers can provide continuous monitoring of vital signs and thus have the potential to become a complementary, mobile and effective tool for early detection of patient deterioration and post-operative complications.

**Methods:**

To evaluate potential implementations in acute care setting, we included 36 patients after moderate to major surgery in a recent randomised pilot trial to compare the performance of vital sign monitoring by three different fitness trackers (Apple Watch 7, Garmin Fenix 6pro and Withings ScanWatch) with established standard clinical monitors in post-anaesthesia care units and monitoring wards.

**Results:**

During a cumulative period of 56 days, a total of 53,197 heart rate (HR) measurements, as well as 12,219 measurements of the peripheral blood oxygen saturation (SpO_2_) and 28,954 respiratory rate (RR) measurements were collected by fitness trackers. Under real-world conditions, HR monitoring was accurate and reliable across all benchmarked devices (r = [0.95;0.98], *p* < 0.001; Bias = [−0.74 bpm;−0.01 bpm]; MAPE∼2%). However, the performance of SpO_2_ (r = [0.21;0.68]; *p* < 0.001; Bias = [−0.46%;−2.29%]; root-mean-square error = [2.82%;4.1%]) monitoring was substantially inferior. RR measurements could not be obtained for two of the devices, therefore exclusively the accuracy of the Garmin tracker could be evaluated (r = 0.28, *p* < 0.001; Bias = −1.46/min). Moreover, the time resolution of the vital sign measurements highly depends on the tracking device, ranging from 0.7 to 117.94 data points per hour.

**Conclusion:**

According to the results of the present study, tracker devices are generally reliable and accurate for HR monitoring, whereas SpO_2_ and RR measurements should be interpreted carefully, considering the clinical context of the respective patients.

## Introduction

Despite decades of technological advancements in health care, nearly 8% of the fatal casualties worldwide are still associated with an operation, especially during the post-operative period. Extrapolated to the U.S., such ‘post-operative mortality’ ranks third in the leading causes of death.^1,2^ This exemplifies the urgent need for enhancements in monitoring patients during their post-operative period.

Nowadays, the vital parameters of post-operative patients are monitored intensively for a comparatively short period of time in the post-anaesthesia care unit (PACU) and/or at an intensive /intermediate care unit (ICU/IMC). For the remaining inpatient hospitalisation at peripheral wards, the monitoring of vital parameters is carried out manually by nurses, therefore routinely restricted to merely episodic and sporadic measurements as the measurements are time-consuming.^3^ This results in a limited assessment of the patient's physiological state, which may hinder an early detection of deterioration during the unobserved phases,^4^ thus leading to an avoidable delay for timely interventions. The most meaningful vital parameters for hospitalised patients include, but are not limited to, heart rate (HR), blood oxygen saturation (SpO_2_) and respiratory rate (RR).^5^ In contrast, the continuous monitoring of vital parameters may enable an early and reliable detection of deterioration^6^ or post-operative complications, consequently lowering the ‘failure to rescue’ rate.^7^

However, state of the art clinical standard bedside monitors used in perioperative or intensive care units, seems unsuitable for tackling the challenge of continuous monitoring, as it generates disproportionate cost and as an even more important reason severely impairs the mobility of patients. With this in mind wrist-based consumer-grade fitness trackers based on the innovative technology of reflective pulse oximetry employing photoplethysmography (PPG) sensors^8^ could offer an auspicious possibility of continuously monitoring vital signs at manageable costs and without impairing the mobility of patients. In short, PPG derives vital parameters based on the intensity of multi-spectral light reflected by the tissue, which is altered by shifts in the absorption spectra caused by physiological processes.^9^

Recently, a pilot study has demonstrated first promising results^10^ on continuously monitoring vital signs by reflective pulse oximetry. However, before this technology can be used for automated, continuous, non-invasive ward monitoring in routine clinical practice, accurate validation of the performance and reliability of reflective pulse oximetry measurements is paramount, and additional challenges must also be addressed.^11^ Previous validation studies of fitness trackers have shown encouraging results for measuring different vital signs, but prevailingly were limited to on-demand measurements under controlled or even under laboratory conditions.^8,12,13^ Longitudinal validation studies examining the performance of automated vital sign measurements by fitness trackers under real-world conditions in hospitalised patients are essential but have yet to be conducted.

Motivated by these shortcomings, we designed a longitudinal, randomised pilot study, in order to benchmark the measurement accuracy and reliability of three popular consumer-grade fitness trackers. Accordingly, we intended to examine the available HR, SpO_2_ and RR background readings of the Apple Watch 7, the Garmin fenix 6 pro and the Withings ScanWatch in post-operative hospitalised patients and compared them in detail with the corresponding clinical standard devices.

## Methods

### Study design

The recruitment for the present prospective, observational and randomised pilot study started in May 2022 at the Department of Anaesthesiology, Intensive Care, Emergency and Pain Medicine at the University Hospital Würzburg, Germany. The investigated devices were randomised (i.e. letter-based randomisation) among the study participants. All aspects of the study protocol are in accordance with the ethical, moral and scientific principles as defined by the declaration of Helsinki (2013, Fortaleza), as well as with the guidelines of good clinical practice. The ethics committee of the University of Würzburg in Germany approved the study protocol prior to the recruitment of the trial (ref. no. 145/21_c). The results presented are part of the ‘Monitor’ trial, which has been registered at clinicaltrials.org (accession no. NCT05418881). The study was designed, conducted and analysed without the financial support or any other contribution by industrial partners, to avoid a potential conflict of interests.

The primary objective of our trial was the evaluation of the HR, SpO_2_ and RR measurement accuracy by consumer-grade fitness trackers with respect to the respective clinical standard. As clinical standards, we defined a 3-lead ECG for HR, transmissive pulse oximetry for SpO_2_, and derived respiratory rate monitoring based on impedance pneumography for RR. For deciding on a ‘clinical acceptable performance’, the following thresholds were adopted: (a) HR with an absolute error <5 bpm, according to the ANSI/AAMI requirements for ‘cardiac monitors, HR metres and alarms’;^14^ (b) SpO_2_ with a root-mean-square error (RMSE) ≤4% in accordance with the ISO 80601-2-61:2019^15^ and (c) RR with a bias of ±3 breaths/min according to established literature.^16^ The secondary endpoint of our trial was defined to assess data quantity as by valid measurements per hour.

Although the a priori estimation of case numbers has limited validity, we calculated based on Cohen's model a case number per group of 9.3, when assuming a Pearson correlation coefficient (r) of 0.78, alpha 0.05 and beta 0.8. Assuming further, a dropout rate of approximately 20%, we decided to include 12 subjects per group in the study.

### Study procedure

Prior to surgical procedure, we screened patients (≥18 y.o.) scheduled for elective moderate or major surgery (according to ESC/ESA guidelines^17^) without planned post-operative invasive ventilation. Exclusion criteria were defined as critically ill patients (defined as ASA V), patients with infectious diseases (for ensuring hygienic safety), patients unable to provide written informed consent (i.e. patients who cannot understand/read the patient information sheet in German or who are incapable of giving written informed consent), as well as patients that already had participated in the Monitor trial before. To guarantee the patients’ safety, known nickel, latex or silicone allergies as well as extensive pathological skin lesions on the forearm were considered as exclusion criteria. All participating patients provided their written informed consent before the surgical procedure. Patients requiring an unplanned post-operative ventilation or surgical procedure terminated prematurely were secondarily excluded.

After the surgical procedure, the participants were randomly assigned to one of the three investigated consumer-grade fitness trackers: (a) the Apple Watch 7, (b) the Garmin Fenix 6 pro or (c) the Withings ScanWatch. Randomisation was implemented by a paper-based method, employing sealed letters. Before the start of the study, an independent member of the study team, not involved in the conduction or statistical analysis, prepared randomisation envelopes. These sealed letters contained a consecutive number and the assignment to one of the study groups following the ‘sealed envelope tool for a blocked randomisation list in clinical trials’ model. Subsequently, the envelopes were opened in the designated sequence for randomisation, noting the respective randomisation group and the letters were archived in the investigator side files.

According to the clinical standard operating procedures, patients were monitored at the PACU and, if necessary, monitoring was continued at the intermediate care unit or the intensive care unit, using the IntelliVue X3 (Philips Healthcare, Eindhoven, Netherlands) and a bed-side monitor (MX750, Philips Healthcare, Eindhoven, Netherlands). These devices provide recording continuous 3-lead electrocardiography (ECG) and transmissive pulse oximetry (FAST Sensor M1191B, Philips Healthcare, Eindhoven, Netherlands). A patient's participation in the present study had no influence on the decision of continued monitoring in a monitoring ward. Immediately after the surgery, each study participant was additionally equipped with one of the fitness trackers, as assigned by the previous randomisation, which was attached to the wrist by our trained research staff according to the manufacturer's instructions. The trackers then were used by the patients during their entire hospital stay. Throughout the entire study period, the investigators daily visited the patients to check the correct positioning of the trackers, to ensure the network connectivity of the smartphones and to recharge the batteries of trackers and smartphones whenever necessary.

### Data acquisition

Before first-patient-in, anonymised user accounts were set up in the respective application of each fitness tracker manufacturer. For all devices, the measurement of HR, SpO2 and RR was activated with the highest possible time resolution. Standard attributes, including sex, age, BMI (including height and weight), heart rhythm, wrist circumference, skin tonality on the Fitzpatrick scale, hair density according to the scale of an in-house categorisation (0 = no forearm hair, 1 = minimal, 2 = moderate, and 3 = extensive hair density on the forearm) and type and length of surgery were recorded for each participant.^8^ The duration of a surgery was defined as the time span from the beginning to the end of anaesthesia. Furthermore, the Charlson comorbidity score, the ASA score and Barthel score were documented in order to assess pre-existing diseases and the overall physical state.

The software of all benchmarked devices was updated to the respective most recent version prior to the start of recruitment. Afterwards, automated software updates were deactivated where possible, to not interfere with results collected in the course of the trial. In addition to the investigated fitness trackers, all study participants needed to be equipped with a smartphone (i.e. an iOS-based iPhone 6 for the Apple watches, and a Nokia G20 Android-based phone for all other tracker devices) required for maintaining the Bluetooth connection to the fitness tracker and transferring all collected data via WiFi. To collect the vital parameter readings from the trackers, the corresponding manufacturer's default application (Apple Health, Garmin connect and Withings healthmate) for the given operating system has been installed on the smartphone. In this setup, measurements by the fitness trackers were stored on the smartphone or sent via WiFi to the cloud-service of the corresponding manufacturer for further processing and storage. Finally, vital signs were requested from the manufacturer's cloud employing the ‘application programming interface’ (API) of each manufacturer-specific ‘representational state transfer’ (REST) interface or downloaded from the smartphone, and stored at our department.

The vital sign measurements by the clinical standard Philips Monitoring are routinely processed and stored at our clinic's facilities. These data are collected by our Patient Data Management System with a time resolution of one measurement per 30 s. Moreover, all collected data points are time-stamped, which enables us to subsequently compare measurements taken at the same time by different devices.

### Statistical analyses

All data analyses were performed employing the R platform (v4.2.0). For comparing demographic data of patients assessed by one of the benchmarked trackers, we computed the standard statistical indicators as the quartiles, the interquartile range (IQR) and Fisher's exact test statistics by the corresponding R kernel functions.^18^ We defined a *p *< 0.05 for statistical significance. Beyond these, the dplyr package v1.1.2 was employed for calculating one-way analysis of variance (ANOVA) test statistics on the comparison of continuous variable distributions in patient attributes (i.e. the height, the weight, the BMI, the wrist circumference and operation time).^19^

**Figure 1.**
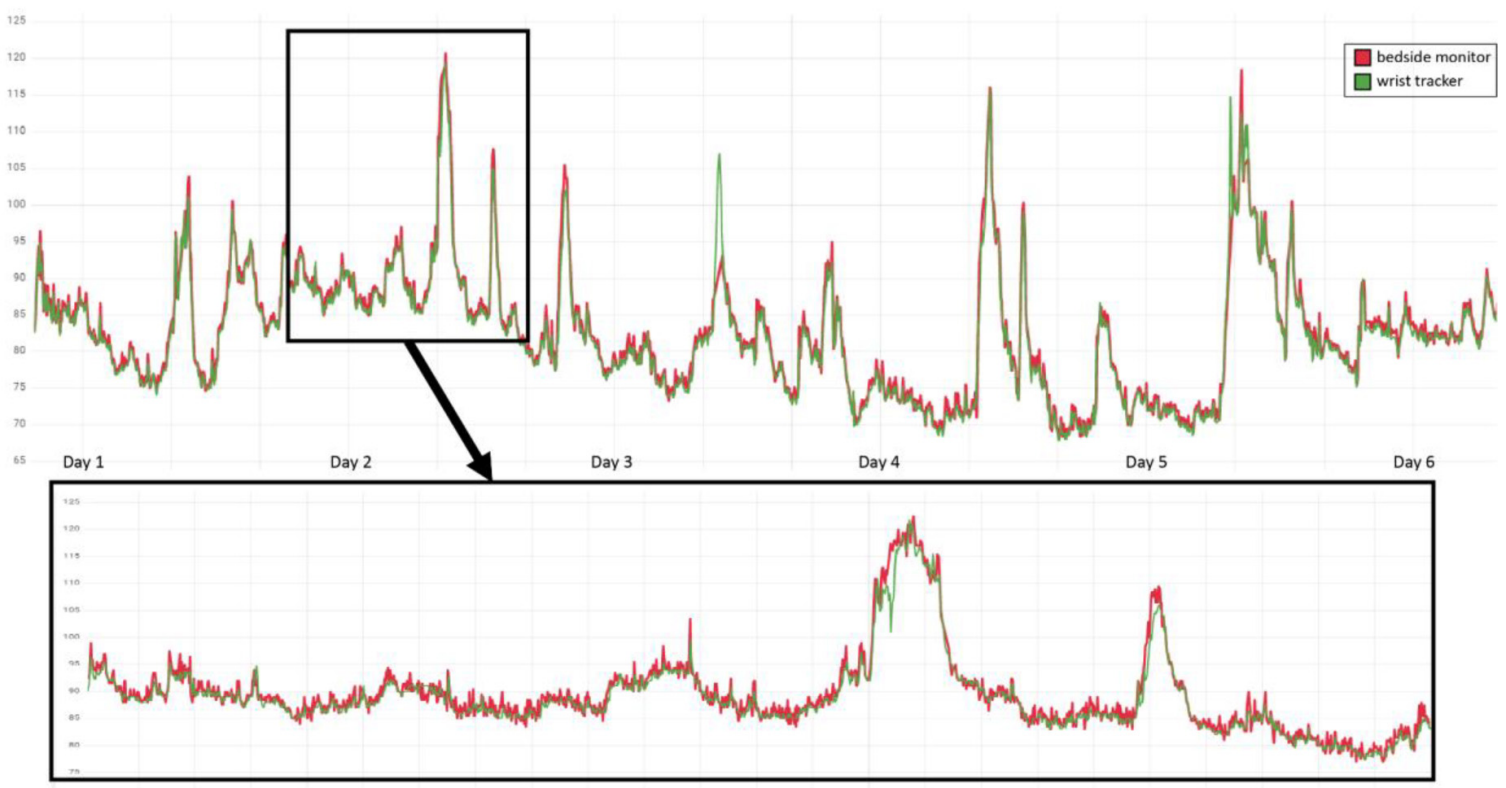
Example QC by visual inspection of HR measurements in one of the patients. Time points of measurement are ordered along the *x*-axis, with reference measurements by the bedside monitor depicted in red and accordingly the tracker measurements in green, and the *y*-axis resolves the correspondingly measured HR in bpm. The upper panel provides an overview of the measurements during the entire 6 day hospitalisation at PACU and IMC of the patient, and the lower panel a 24 h zoom-in.

Figure 1 depicts an example of reference (red line) and tracker (green line) HR measurements. During quality control (QC), we visually inspected the synchronisation of both of the compared measurement time series, in order to exclude systematic shifts as could, for instance, be introduced by time zone mismatches or missettings of the clock in one of the compared devices. Subsequently, we matched each tracker measurement *q* (green) to its closest reference measurement *p* (red), excluding measurement pairs (*p,q*) that were taken >30 s from each other and also preventing from assigning the same reference or tracker measurement multiple times.

Considering *i* paired measurements (*p_i_*,*q_i_*), we further removed outliers with an observed deviation |*p_i_* − *q_i_*| of >30 bpm (HR), > 10% (SpO_2_), and, respectively, >15 min^−^1 (RR) and labelled them as QC failures. These thresholds were defined based on clinical judgement. We then employed the ggplot2 (v3.3.6) library for visualising the scatter and Bland–Altman diagrams. Based on the Metrics (v0.1.4) package,^20^ we calculated the mean absolute error (MAE) by *mean*(|*p_i_* − *q_i_*|), the (square) RMSE by *sqrt*(*mean*((*p_i_* − *q_i_*)2)) and the mean absolute percentage error (MAPE) by *mean*(|*p_i_* − *q_i_*| × 100/q).

Following the analysis proposed by Bland and Altman,^21^ we employed scatter plots to stratify the observed error (*p_i_* − *q_i_*) by the arithmetic average of the predicted and reference values (*p_i _*+ *q_i_*)/2. The bias *B* of the benchmarked measurements was estimated by the arithmetic average of all these real errors mean(*p_i_* − *q_i_*), and the upper as well as the lower limit of agreement (*LoA*) were obtained by an offset of twice the corrected sample standard deviation 2 × *sd*(*p_i_* − *q_i_*). Eventually, the confidence intervals of *B* and *LoA* were determined assuming a Student's *t*-distribution, by employing the R function *qt*().^22^

## Results

### Overview of the cohort

**Figure 2.**
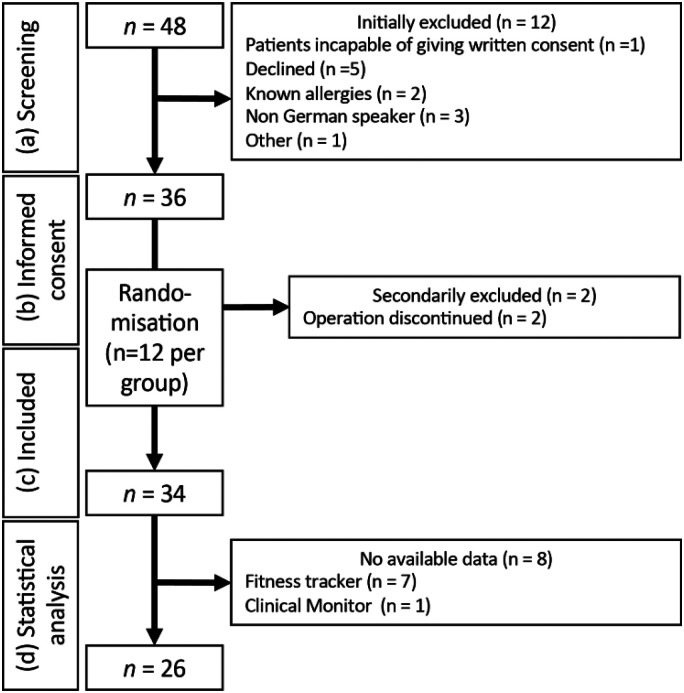
Flowchart of the patient screening, recruitment and statistical analysis.

A total of 48 patients were screened, of whom 36 patients provided written informed consent. After randomisation, 34 patients were successfully enrolled in the study (see Figure 2). Of these, 12 underwent urological surgery (nephrectomies, cystectomies and cystoprostatectomies) and 22 non-cardiac thoracic surgery (segmental resection of lung). Due to the unavailability of data from 7 fitness trackers (6× Withings and 1× Apple) and one clinical monitor, finally 26 patients were included in the analysis. This results in a group size of *n* = 10 for Apple, *n* = 12 for Garmin and *n* = 4 for Withings.

### Patient characteristics

The median age of the prevailingly Caucasian (Fitzpatrick-scale 2 [IQR 1]) cohort was 65.5 years (IQR 12.25), with a BMI of 27 kg/m^2^ (IQR 6.65) and constituted by more males than females (53.85% vs. 46.15%). The majority of the patients exhibited sinus rhythm (92.3%) and suffered from mild to severe systemic diseases (ASA median = 2, IQR = 1). The Charlson comorbidity index was 3 (IQR 2.75), and the patients presented fully preserved ability to cope with everyday life (Barthel index 100, IQR 0). The analysed metadata between the groups differed significantly in gender distribution, and subsequently also in BMI and wrist circumference. A detailed overview of patient attributes by randomisation group is shown in Table 1.

**Table 1.** Metadata analysis of patients in the study.

	All	Apple	Garmin	Withings	*p*
Group size (*n*)	26	10	12	4	0.0502
Age (years)	65.50 (12.25)	63.00 (13.50)	67.00 (16.25)	65.50 (6.00)	0.2925
Sex (% female)	46.15	70.00	8.30	100.00	**0**.**0004**
BMI (kg/m^2^)	27.00 (6.65)	29.45 (6.13)	26.85 (4.40)	21.30 (1.70)	**0**.**0152**
Wrist circumference (cm)	18.00 (2.50)	18.50 (2.38)	18.00 (1.00)	16.00 (0.50)	**0**.**0248**
Height (cm)	173.00 (13.75)	170.00 (9.75)	178.50 (8.25)	163.00 (4.25)	0.7950
Weight (kg)	83.00 (25.75)	83.00 (21.50)	86.50 (19.50)	60.50 (11.50)	0.0568
ASA	2.00 (1.00)	2.00 (1.00)	2.00 (1.00)	2.50 (1.00)	0.8748
Fitzpatrick	2.00 (1.00)	2.5 (1.00)	2.00 (1.00)	2.50 (1.50)	1.0000
Hair density	1.00 (2.00)	1.0 (1.00)	2.00 (1.00)	0.00 (0.25)	0.0651
Sinus rhythm (% sinus)	92.31	90.00	91.67	100.00	1.0000
Barthel index	100.00 (0.00)	100.00 (0.00)	100.00 (0.00)	100.00 (0.00)	1.0000
Operation time (min)	283.00 (203.00)	283.00 (207.25)	297.50 (194.75)	306.50 (182.25)	0.9544
Charlson comorbidity index	3.00 (2.75)	3.00 (3.75)	3.50 (2.50)	4.00 (2.75)	0.8492

For each variable (lines, name of the variable in column 1), the distribution of values in each (sub-)cohort of the patients (i.e. columns 2–5: all patients, as well as patients assessed with the Apple, the Garmin and the Withings tracker) is characterised by either the total number of instances (i.e. variable ‘Group size’) or by the fraction of instances (in [%]) in one of the binary classes (i.e. variables ‘Sex’ and ‘Sinus rhythm’), or by the median and, respectively, the interquartile range (in parentheses) for the remaining variables. Significant influences by categorical factors (i.e. Group size, Sex, ASA, Fitzpatrick, Hair density, Sinus rhythm, Barthel index and Charlson comorbidity index) are estimated each by a Fisher exact test, whereas the significant influences by metric factors (i.e. Age, BMI, Wrist circumference, Height, Weight and Operation time) are estimated by an ANOVA test. *p*-values (last column) below the significance level of 0.05 are shown in bold.

**Table 2.** HR monitoring benchmarking indicators for each of the investigated devices.

	Apple	Garmin	Withings
m	8057	43874	1266
QC failure rate (%)	0.07	0.02	2.16
Time resolution (data points per hour)	14.43	117.94	5.58
MAE (bpm)	1.67	1.64	1.87
MAPE (%)	2	2	2
MSE	7.23	7.02	10.23
Bias (bpm) (LoA)	−0.73 (−5.9;4.45)	−0.74 (−5.83;4.35)	−0.01 (−6.41;6.39)
R	0.98	0.97	0.95
Rc	0.98	0.97	0.95

m: measurements; QC: quality control; MAE: mean absolute error; MAPE: mean absolute percentage error; MSE: mean squared error; LoA: limits of agreement; r: Pearson correlation coefficient; rc: Lin's concordance coefficient; bpm: beats per minute.

### Benchmarking

During the study period, a total of 1352.12 h reference measurements and 1157.24 h of tracker measurements were collected, intersecting by 85.59% in time. Within this time overlap, overall 53,240 pairs of data points could be matched for benchmarking HR, as well as 12,662 pairs for SpO_2_ and 29,364 pairs for RR. Purging QC failures (cf. thresholds in Statistical analyses section) yielded 53,197 (99.92%) data pairs for HR, 12,219 (96.50%) data pairs for SpO_2_ and 28,954 data pairs (98.60%) for RR.

#### Heart rate

Measurement pairs with a difference of >30 beats/min removed during QC were rare across the cohort, with Withings showing the highest QC failing rate of 2.16%, followed by Apple with 0.07% and Garmin with 0.02% (see Table 2). In the end, 558.4 h (i.e. 8057 data pairs) for Apple, 372 h (i.e. 43,874 data pairs) for Garmin and 226.84 h (i.e. 1266 data pairs) for Withings could be included to analyse the HR measurement accuracy. Extrapolated to the number of hours of use of the devices, this result in a quantity of successful measurements per hour of 14.43 h^−1^ for Apple, 117.94 h^−1^ for Garmin and 5.58 h^−1^ for Withings.

**Figure 3.**
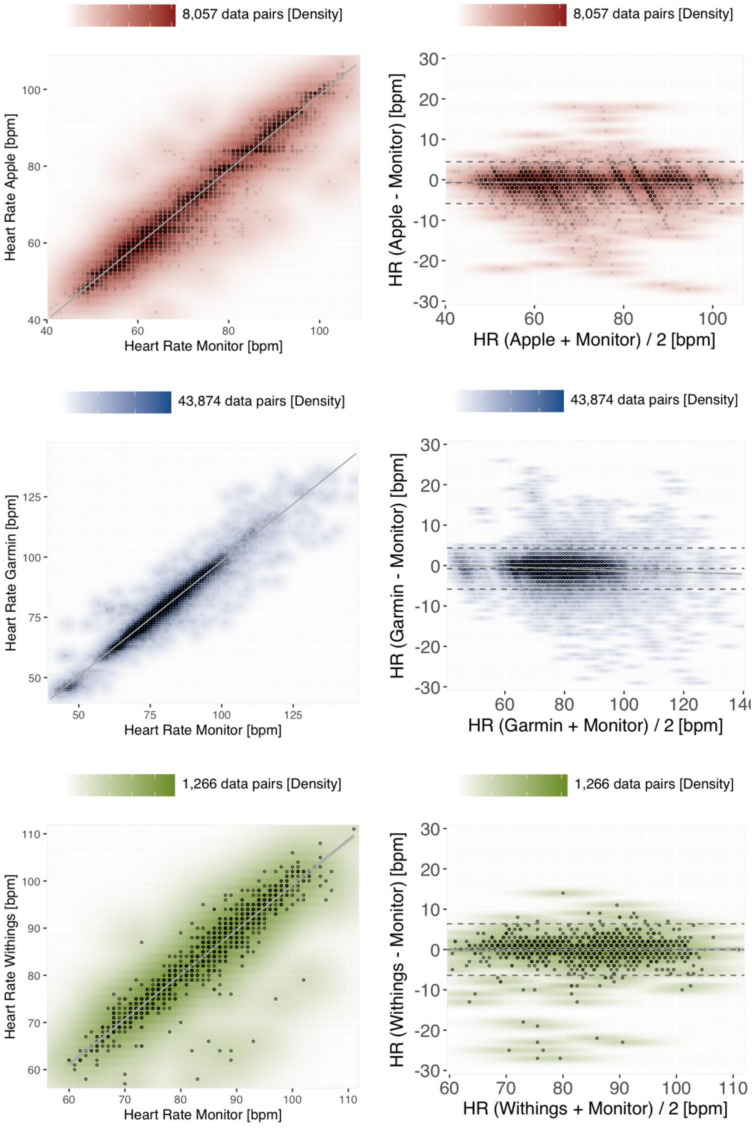
Density scatter plots (left panels) and Bland–Altman plots (right panels) to assess measurement accuracy for HR. The paired reference-tracker measurements are colour-coded according to the benchmarked devices, with Apple in red, Garmin in blue and Withings in green. A scale orienting the density of data points coinciding at the same coordinates is provided above each diagram. Left panels: The *x*-axis of the scatter plots shows the HR measurements (bpm) of the reference method in comparison to the corresponding measurements (bpm) of the benchmarked tracker device along the *y*-axis. The grey line visualises the linear regression within a 95% confidence interval. Right panels: The *y*-axis of the Bland–Altman plots shows the difference between tracker and reference measurements, stratified by the average between both measuring methods on the *x*-axis. The bias and the upper/lower limits of agreement are depicted as dashed grey horizontals, the linear regression within 95% of confidence as a solid grey line.

Figure 3 shows that the best correlation of HR measurements was observed for Apple, reaching the requirements of the clinical gold standard (r = 0.98; *p* < 0.001), followed by Garmin (r = 0.97; *p* < 0.001) and Withings (r = 0.95; *p* < 0.001). The empirically determined mean square error (MSE) was comparable for Apple and Garmin measurements (7.23 vs. 7.02), albeit higher for Withings measurements (10.23).

On average, all the investigated devices slightly underestimated the reference measurements, with the smallest absolute bias for Withings −0.01 bpm [−0.19;0.17], followed by Apple −0.73 bpm [−0.78;−0.67] and Garmin −0.74 bpm [−0.76;−0.71]. The LoA differed merely marginally between Apple [−5.9;4.45], Garmin [−5.83;4.35] and Withings (−6.41;6.39). Reassuringly, very similar indicators are estimated under a model considering mixed linear effects of paired replicate measurements^23^ obtained in each of the patients (Supplemental Figure 1).

#### Oxygen saturation

As compared to HR readings, the fitness trackers exhibited considerably less SpO_2_ measurements in our trial. Therefore, also the time spans overlapping between reference and tracker measurements are substantially shorter, with 280.90 h for Garmin, 146.79 h for Withings and merely 118.35 h for Apple. After removing outliers, a total of 11,396 data pairs for Garmin, 424 data pairs for Withings and 399 data pairs for Apple could be included for analysis. Considering these overlap times, the empirically estimated data quantities for Garmin yield 40.57 data points per hour, 3.37 h^−1^ for Apple and 2.89 h^−1^ for Withings. However, based on the total time of wearing the trackers, estimates rank differently, with 0.7 h^−1^ for Apple and 1.87 h^−1^ for Withings. Also the QC failing rate are significantly higher for SpO_2_ measurements as compared to the HR measurements: here, 2.21% of the Apple measurements exhibited a deviation >10% from the reference method, correspondingly 2.94% of the Garmin measurements and up to 17.35% of the Withings measurements.

**Figure 4.**
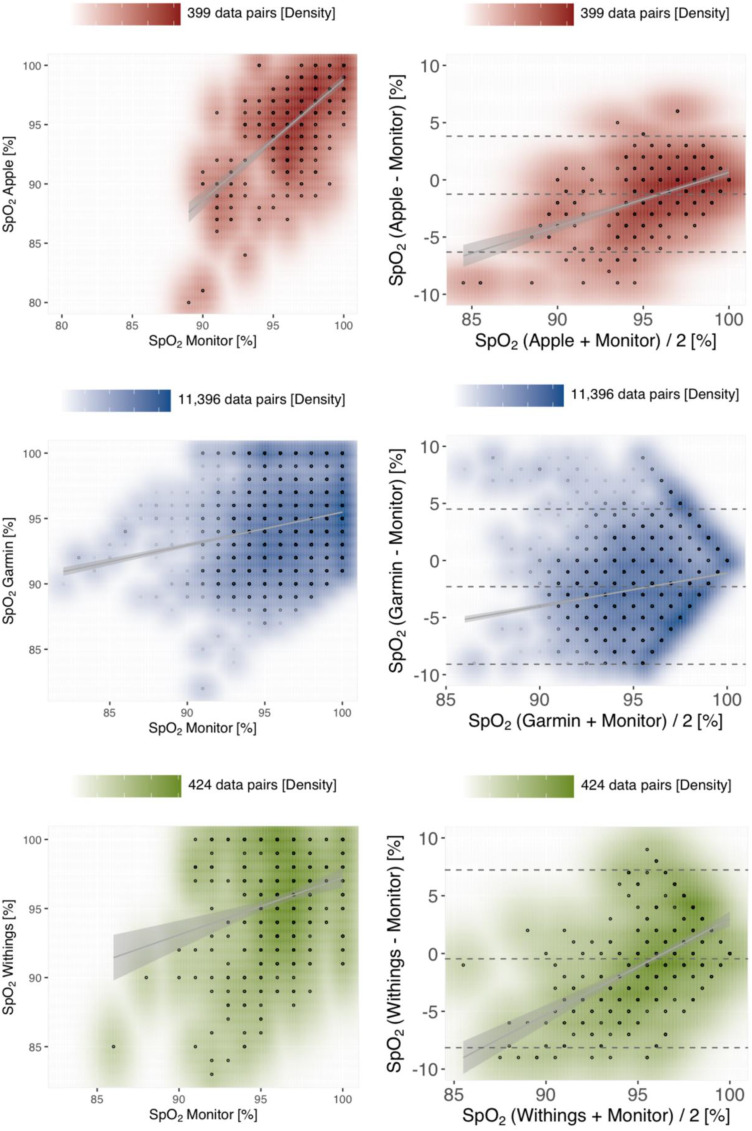
Density scatter plots (left panels) and Bland–Altman plots (right panels) assessing the measurement accuracy of tracker devices for SpO_2_. The plot layout (solid grey lines/areas visualising the linear regression within a 95% confidence interval, and dashed horizontal lines for the bias and, respectively, the limits of agreement), density scales above each plot, and the colour-codes are preserved from Figure 3 (Apple in red, Garmin in blue and Withings in green). m: measurements; RMSE: root mean square error; LoA: limits of agreement; r: Pearson correlation coefficient; rc: Lin's concordance coefficient.

All of the investigated devices slightly underestimated the SpO_2_ value assessed by the reference method (see Figure 4). Withings exhibited the smallest absolute bias with −0.46% (with LoA −8.15;7.23), whereas Garmin measurements had the largest absolute bias with −2.29% (LoA −9.09;4.5). Apple achieved the lowest RMSE in the benchmark of 2.82 and Garmin the highest RMSE of 4.10 (see Table 3). These results are furthermore reflected in part by the Pearson correlation coefficients of *r *= 0.68 (*p* < 0.001) for Apple as compared to *r *= 0.21 (*p* < 0.001) for Garmin.

**Table 3.** SpO_2_ benchmarking indicators for each of the investigated devices.

	Apple	Garmin	Withings
m	399	11,396	424
QC failure rate (%)	2.21	2.94	17.35
Time resolution (data points per hour)	3.37	40.57	2.89
RMSE	2.82	4.10	3.87
Bias (LoA)	−1.25 (−6,31;3.81)	−2.29 (−9.09;4.5)	−0.46 (−8.15;7.23)
r	0.68	0.21	0.24
rc	0.58	0.15	0.2

m: measurements; QC: quality control; RMSE: root mean squared error; LoA: limits of agreement; r: Pearson correlation coefficient; rc: Lin's concordance coefficient.

#### Respiratory rate monitoring

**Figure 5.**
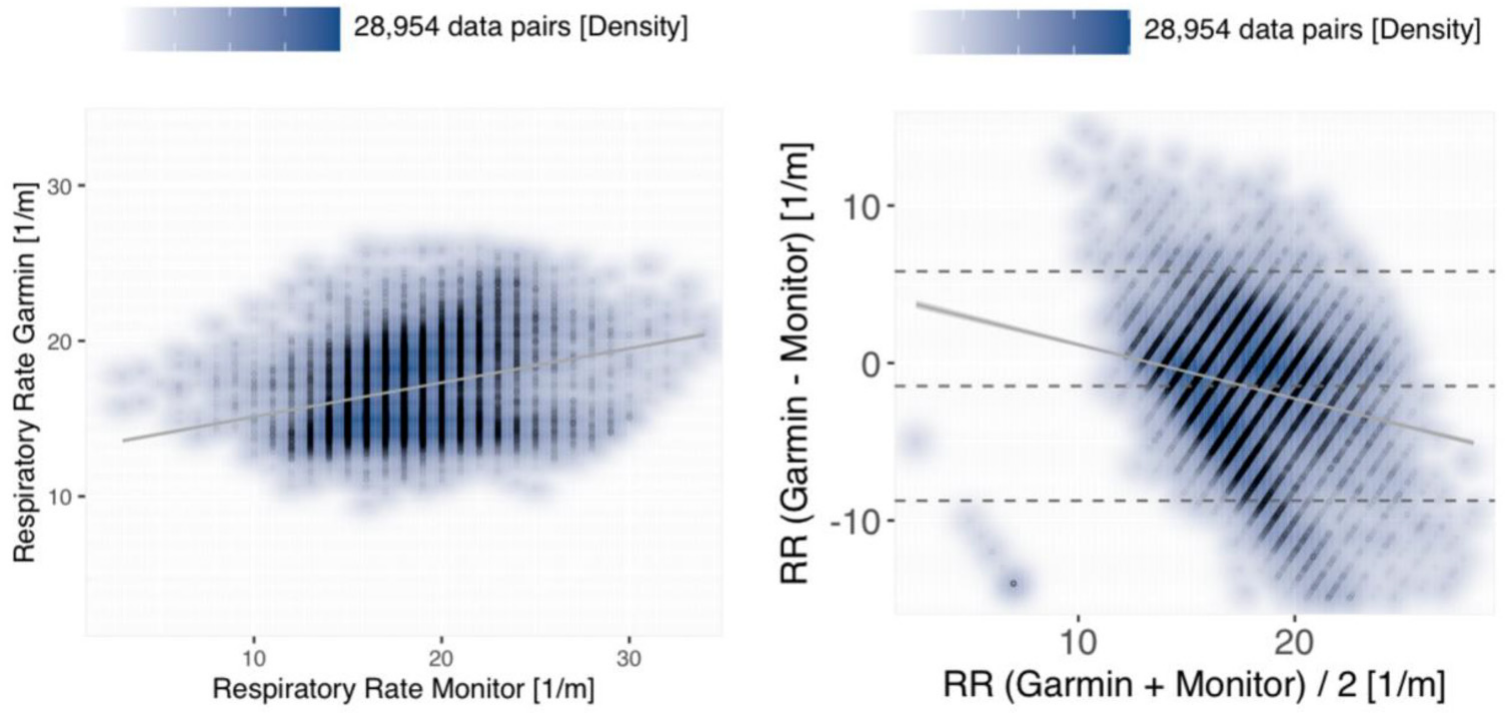
Density scatter plot (left panel) and Bland–Altman plot (right) for the respiratory rate measurements obtained by the Garmin tracker. Visualisations as introduced before (cf. Figures 3 and 4).

Although all of the investigated tracker devices are capable of monitoring the RR in theory, our study yielded readings exclusively from the Garmin device. During an overlap time of 353.89 h with the reference measurements, a total of 29,364 Garmin data pairs could be obtained. After employing the QC defined as >15/min deviance from the reference, a total of 28,954 (98.6%) of the data pairs were submitted to statistical analysis. The benchmarked data points correspond to a temporal resolution of 81.82 valid data points per hour collected by the tracker. However, the observed correlation between the Garmin RR measurements and the reference was relatively poor (r = 0.28; *p* < 0.001), albeit exhibiting a comparatively low bias of −1.46/min (LoA −8.74;5.82) (see Figure 5).

## Discussion

The aim of our study was to benchmark the continuous monitoring of different vital signs – namely the HR, SpO_2_ and RR – employing consumer-grade fitness trackers. Our motivation was not to propose these devices as a prospective alternative to existing systems at monitoring wards, but rather to assess a potential implementation of the investigated fitness trackers as auxiliary surveying in hospital scenarios, predominantly for the post-operative monitoring of patient vital sign parameters. In this context, we benchmarked the quality, that is, the measurement accuracy, but also the quantity of the data obtained, that is, the time resolution of the measurements.

According to our results, the quality of the measurements largely depends on the vital parameter assessed, and to a minor extent also on the investigated device. In the studied cohort, all the trackers of different manufacturers demonstrated notably accurate measurements of the HR, with an overall bias of <1 bpm and an overall Pearson correlation coefficient of >0.95. In our study, only a minority part of 0.08% of the collected tracker measurements differed substantially (i.e. >30 bpm) from the ground truth references. Reassuringly, our findings are consistent with our previous observations (r > 0.95 and bias <1.21 bpm) in a study on resting patients under controlled conditions,^8^ and also in agreement with complementary literature.^24^ Employing our predefined thresholds (i.e. absolute error <5 bpm), all devices performed with a clinical acceptable measurement accuracy.

In contrast to the HR, the ability of monitoring SpO_2_ is substantially inferior for all of the investigated tracker devices. The number of QC failures (i.e. the number of tracker measurements with >10% deviation from the reference) are overall >40× higher in the SpO_2_ benchmark as compared to the HR benchmark – including almost one out of five SpO_2_ measurements by the Withings tracker. However, the biases observed for SpO_2_ measurements seem comparatively low, ranging from −2.29% [−2.36;−2.23] for Garmin to −0.46% [−0.83;−0.09] for Withings. Our observations are supported by complementary studies based on the Apples Watch 6 and 7 models,^12,25^ where similar RMSE estimates of 3%–3.5% for the SpO_2_ measurements in healthy subjects or patients with chronic obstructive pulmonary disease are reported.

Employing the accuracy requirement of RMSE ≤ 4 set by the ISO standard (80601-2-61:2019),^15^ in our study, Apple (RMSE = 2.82%) as well as Withings (RMSE = 3.87%) would pass this threshold, whereas Garmin (RMSE = 4.10%) slightly fails. However, it is to be noted that our study does not meet pre-requirements of the ISO standard, according to which SpO_2_ parameters need to cover the range 70%–100%, equally distributed amongst the participants. Despite tolerable RMSE indicators, all of the benchmarked tracker devices exhibit only a poor (Garmin r = 0.21; Withings r = 0.24) to moderate (Apple r = 0.68) correlation with the reference SpO_2_ measurements. These findings seem counterintuitive considering that the Apple as well as the Withings fitness tracker were cleared by the Food and Drug Administration for SpO_2_ monitoring. Comparing our evaluations to a study on the SpO_2_ measuring accuracy of the ScanWatch tracker, conducted by the Withings manufacturer, we observe similar QC failure rates (17.35% vs. 19.4% on average of both hands), a higher but comparable RMSE (3.87% vs. 3.0%), however, a substantially lower Pearson correlation coefficient (0.24 vs. 0.95).^26^

On the other hand, comparing our results to the indicators reported on the SpO_2_ measurement accuracy of the Apple Watch, published in a white paper by Apple,^27^ we find comparable values for the RMSE (2.8% vs. <2.2%) as well as for the linearly regressed model (m,t = 1.02,3.00 vs. 0.959,3.906). However, it needs to be considered that the RMSE is a non-normalized indicator and therefore captures numerically the range of the condensed measurements and also the measurement errors, respectively. RMSE indicators therefore have a limited comparability across studies and our results cannot be put directly on scale with the latter studies, which have been carried out on cohorts in accordance with the ISO standard 80601-2-61:2019 (min. SpO_2_ 70%), whereas SpO_2_ reference values in our present trial are >80% (i.e. producing potentially higher RMSE values).

Similar to our observations in the SpO2 benchmark, also RR measurements by the Garmin fitness tracker exhibit bias of −1.46/min (CI = [−1.5;−1.42]), which is within our previously defined limits (i.e. ±3 breaths/min), but a poor correlation (r = 0.28) with the reference measurements. In our study, RR monitoring had also been activated on the other trackers, but no measurements were available from the Apple and the Withings trackers. Even though according to the manufacturer, the Apple Watch and the Withings ScanWatch can display the RR, we unfortunately cannot provide a solid explanation for the failure to output these measurements. Therefore, we also cannot draw any conclusion about the RR measurement accuracy of the latter tracker devices.

Beyond measuring quality, the quantity of measurements obtained by a fitness tracker depends more on the investigated device than on the monitored vital parameter. Garmin provides by far the best metering time period of measurements, with on average at least one HR and RR measurement, and almost one SpO_2_ measurement, being tracked per minute. Of note, for some of the vital parameters, the true output rate of the Garmin device might even be higher, as the data pairs in our benchmark are limited by the metering time period of the reference monitoring measurements, which are available only every 30 s.

The other two trackers in our benchmark, manufactured by Apple and, respectively, by Withings, exhibited in our study a practical metering time period of [0.7;15] data points h^−1^. Concerning the Withings device, we observed a notably lower yield in data quantity than reported by a complementary study, where a specialised and research-adapted firmware version has been employed.^26^ It can be hypothesised that the firmware of the consumer-grade devices we employed out-of-the-box in our study implements specific battery optimisations that prevent from collecting data with high frequency.

Furthermore, this low frequency metering we observed in clinical practice with the Withings but also with the Apple tracker clearly renders both trackers not suitable for monitoring vital signs in crucial scenarios. However, devices with a long metering time period still may be considered in situations that indicate an occasional monitoring of patients. Compared to the presently practised standard on many peripheral wards, that is, vital signs are measured manually only once per shift (every 6–8 h), the use of wearables could complement the monitoring of a patient. In order to contemplate the use of trackers in scenarios along these lines, also the battery operating and recharging time periods need to be evaluated more in detail, which was beyond the scope of our present study.

According to our observations, the utility and usability of fitness trackers for the continuous monitoring of vital signs depends not only on the device employed but also primarily on the vital parameter that is monitored. Concerning surveillance of the HR, all of the benchmarked devices demonstrated a high reliability in their measurements, with Garmin exhibiting the best metering time period in practice. SpO_2_ and RR measurements instead are impacted by a substantially higher degree of variance and bias. Correspondingly obtained readings should therefore be evaluated carefully in the context of the intended use. Our results on the Scanwatch are of limited validity in the general case, because the size of the group analysed is smaller as compared to the other two devices, falling below the a priori assumed sample size.

In a cohort of post-operative patients, the precise measuring of respiratory rate and SpO2 is essential for the prediction of post-operative complications or deterioration of the patients’ health. For example, the respiratory rate must be measured accurately to reliably determine the qSOFA (quick sequential organ failure assessment) score, an essential tool for quickly assessing the medical condition of a patient.^28^ Therefore, inaccuracies in the measurement evidently do not benefit the patient. However, our study rendered particularly the SpO_2_ and RR measurements by fitness trackers are not suitable to substitute clinical standards employed to survey patients in critical conditions, but may provide novel possibilities in conditions where the monitoring of vital signs is recommended at longer time intervals, which currently is realised as single point measurements carried out by healthcare personnel.

## Limitations

Overall, the patients participating in our present study were cumulatively monitored for 1352.12 h by standard clinical monitoring, but we only yielded data from the fitness trackers in 85.59% of this time. One possible explanation for this difference is the time overhead for charging the devices, during which measuring is paused. Furthermore, the overlap of the measuring periods between the reference monitor and the benchmarked trackers also varied for each of the evaluated devices, reflecting the fluctuations in the length of the patients’ stay in a monitoring ward. Another source of tracker measuring failure could consist in the inappropriate attachment of a tracker device to the wrist, although in our study investigators carried out daily patient visits. Although the overall number of measurements in our benchmark is relatively high (∼10^4^ data point pairs), the number of patients that finally could be included in our study is limited (*n* = 36). Despite the device assignment having been uniformly randomised across patients, during analysis we found that no measurement data was available from the manufacturer's cloud repository for six of the Withings patients, which we could not recover, even after personal correspondence with the manufacturer. These missing data created an unexpected variation of cohort size between our benchmarked devices, with the Withings benchmarks falling below our a priori case number estimates, and also distorted the distribution of sex, BMI and also of wrist circumference of the investigated cohorts.

For the RR measuring benchmarks, the impedance pneumography were employed as a reference method, an indirect measuring method based on a 3-lead ECG. Although impedance pneumography is accepted by established clinical standards, the gold standard here would have been to employ CO_2_ monitoring or spirometry. Moreover, based on the herein analysed cohort, no conclusions can be drawn about the ability of the investigated devices to measure extreme vital parameters (e.g. HR <40 or >170/min). Although our study has been conducted under real-world conditions and patients were partially mobilised outside of their beds, overall most of our data were collected from immobilised patients. During the setup of anonymised user accounts on the manufacturers’ websites for the utilisation of Wearables, standardised parameters for height and weight were employed without adjustment for individual study participants. Given that the internal algorithms of the manufacturers remain unpublished, we cannot dismiss a potential emergence of bias. Blinding was unsuitable due to the optically different design (patient blinded) and data structures of the different tracker devices (blinding during analysis). Concerning the analysis, currently related studies report heterogeneous and often also ad hoc defined thresholds for considering a device ‘acceptable for clinical use’. Similarly, the definitions of failed measurements, drop outs, and QC failures are reported inconsistently throughout relevant literature. Efforts to establish a controlled vocabulary and standardised thresholds would be highly needed to ensure the comparability of studies in the future. Based on the study design in an intensive care setting, future studies on the use of comparable devices in peripheral wards are recommended.

## Conclusion

Each of the examined devices demonstrated a high degree of reliability and accuracy in monitoring the HR. However, it is advisable to take measurements of SpO2 by all manufactures and of the respiratory rate of Garmin with care, considering the clinical context of the application and the status of individual patients.

## Contributorship

Study concept and design by PH, BEW, MS. Data acquisition by PH, SH, KW, VD, AKS, DR, JB, PK, PM. Data analysis by PH, RP, AJ, MS. Data interpretation by PH, MS. Supervision by RP, PK, PM. Writing of the initial manuscript draft by PH, MS. Critical revision by SH, KW, RP, VD, AKS, DR, AJ, JB, PK, PM, BEW.

## Supplemental Material

sj-docx-1-dhj-10.1177_20552076241254026 - Supplemental material for Reliability of continuous vital sign monitoring in post-operative patients employing consumer-grade fitness trackers: A randomised pilot trial Supplemental material, sj-docx-1-dhj-10.1177_20552076241254026 for Reliability of continuous vital sign monitoring in post-operative patients employing consumer-grade fitness trackers: A randomised pilot trial by Philipp Helmer, Sebastian Hottenrott, Kathrin Wienböker, Rüdiger Pryss, Vasileios Drosos, Anna Katharina Seitz, Daniel Röder, Aleksandar Jovanovic, Jürgen Brugger, Peter Kranke, Patrick Meybohm, Bernd E Winkler and Michael Sammeth in DIGITAL HEALTH

sj-docx-2-dhj-10.1177_20552076241254026 - Supplemental material for Reliability of continuous vital sign monitoring in post-operative patients employing consumer-grade fitness trackers: A randomised pilot trial Supplemental material, sj-docx-2-dhj-10.1177_20552076241254026 for Reliability of continuous vital sign monitoring in post-operative patients employing consumer-grade fitness trackers: A randomised pilot trial by Philipp Helmer, Sebastian Hottenrott, Kathrin Wienböker, Rüdiger Pryss, Vasileios Drosos, Anna Katharina Seitz, Daniel Röder, Aleksandar Jovanovic, Jürgen Brugger, Peter Kranke, Patrick Meybohm, Bernd E Winkler and Michael Sammeth in DIGITAL HEALTH
