# Supplementary material for: Reliability of continuous vital sign monitoring in post-operative patients employing consumer-grade fitness trackers: A randomised pilot trial
Source: Digit Health. 2024 May 13;10:20552076241254026. doi: 10.1177/20552076241254026 (PMC11092531; doi:10.1177/20552076241254026)
Supplement: sj-docx-1-dhj-10.1177_20552076241254026 - Supplemental material for Reliability of continuous vital sign monitoring in post-operative patients employing consumer-grade fitness trackers: A randomised pilot trial [file sj-docx-1-dhj-10.1177_20552076241254026.docx]

# Supplementary Material

## Figure 1. Bland-Altman indicators considering replicates

|  | Apple Watch 7 | Garmin fenix 6 | Withings ScanWatch |
| --- | --- | --- | --- |
| HR | 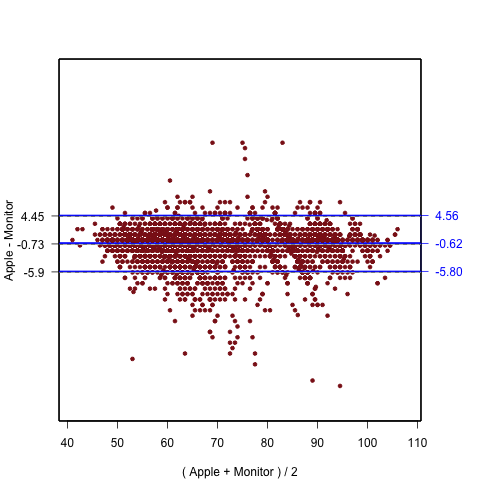 | 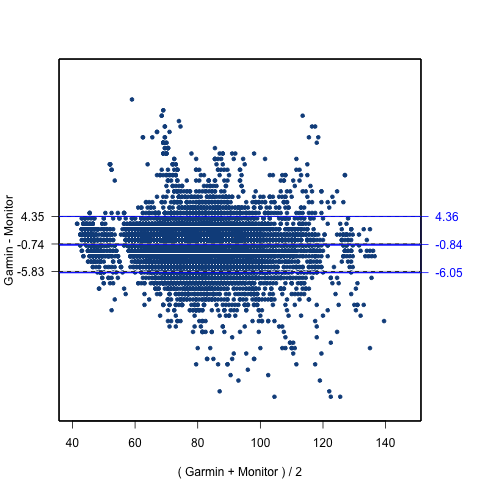 | 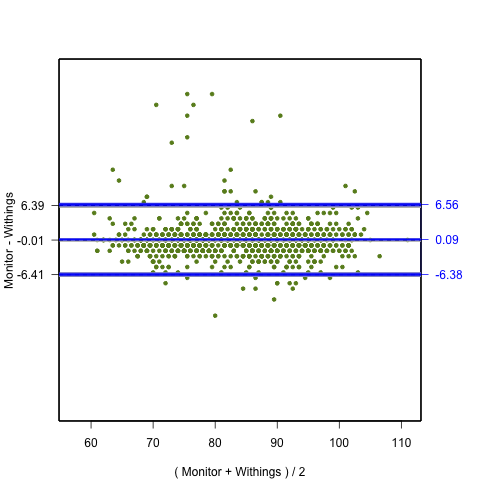 |
| *Sp*O_2_ | 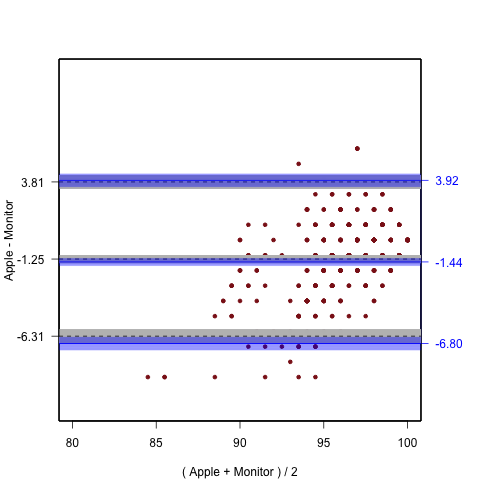 | 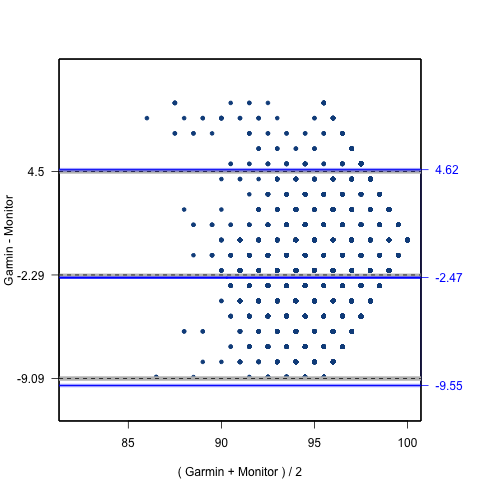 | 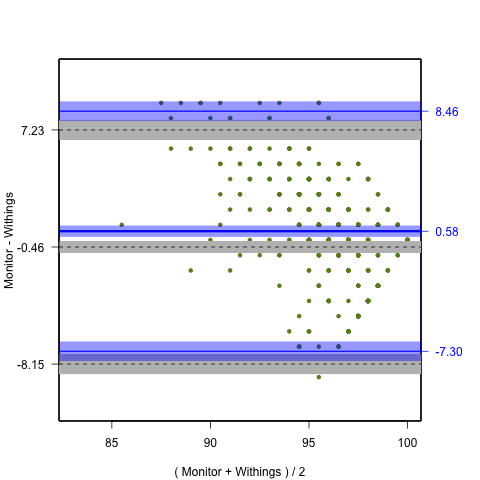 |
| RR |  | 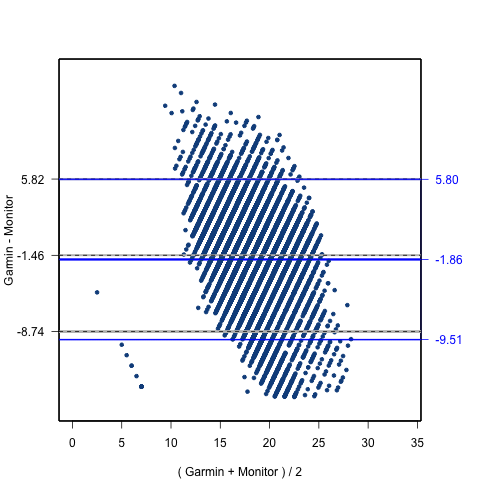 |  |
| **Figure S1: Replicate Measurements.** Diagrams summarising for the investigated datasets the Bland-Altman indicators *Bias*, *Lower* as well as *Upper Limit of Agreement* (horizontal lines) as well as their 95% confidence intervals (semi-transparent horizontal bars), computed when considering the measurement pairs altogether (shown in grey on the left y-axis; cf. Figure 3-5, Table 2 and 3) as compared to estimates by a linear mixed-effect model taking into account possible biases by linked replicate measurements in each of the individuals (shown in blue, right y-axis). HR= Heart Rate [bpm], *Sp*O_2_= peripheral blood oxygen saturation [%], RR= respiratory rate [min^-1^]. | | | |

## 
